# Degradable polymeric vehicles for postoperative pain management

**DOI:** 10.1038/s41467-021-21438-3

**Published:** 2021-03-01

**Authors:** Natasha C. Brigham, Ru-Rong Ji, Matthew L. Becker

**Affiliations:** 1 Department of Chemistry, Duke University, Durham, NC USA; 2 Department of Anesthesiology, Center for Translational Pain Medicine, Duke University, Durham, NC USA; 3 Department of Cell Biology, Duke University, Durham, NC USA; 4 Thomas Lord Department of Mechanical Engineering and Materials Science, Duke University, Durham, NC USA; 5 Department of Biomedical Engineering, Duke University, Durham, NC USA; 6 Department of Orthopaedic Surgery, Duke University, Durham, NC USA

**Keywords:** Drug delivery, Pain management, Biomaterials

## Abstract

Effective control of pain management has the potential to significantly decrease the need for prescription opioids following a surgical procedure. While extended release products for pain management are available commercially, the implementation of a device that safely and reliably provides extended analgesia and is sufficiently flexible to facilitate a diverse array of release profiles would serve to advance patient comfort, quality of care and compliance following surgical procedures. Herein, we review current polymeric systems that could be utilized in new, controlled post-operative pain management devices and highlight where opportunities for improvement exist.

## Abstract (web summary)

Pain management is an extremely important topic both medically and socio-economically. Here the authors offer an overview of the use of degrading polymeric materials for delivery of pharmaceutical agents for pain management and offer a perspective of the future directions of the field.

## Postoperative pain management

Despite wholistic advances in understanding molecular interactions and available pharmaceuticals, postoperative pain management remains challenging^1^. More than 80% of patients who undergo surgery report experiencing acute pain following the procedure with less than half reporting adequate postoperative analgesia^2,3^. The current standard of care for postoperative analgesia is the prescription of orally dosed medication that patients take on an as-needed basis. While convenient, economical and compliant to existing distribution and reimbursement models, this practice requires the patient to determine the frequency of dosing for comfort and healing and can lead to diversion^4,5^. Additionally, oral administration of any active pharmaceutical ingredient (API) results in systemic biodistribution, impacting not only the injured or target tissues, but also all other healthy tissues in the body and is an inefficient use of drug^6,7^. The off-target effects of potent APIs for pain management, especially when misused, are detrimental to patient health and can have significant lasting effects.

A promising solution to postoperative pain management is to implement analgesics or anesthetics into implantable, biodegradable controlled delivery matrices such that the API could be provided locally for a pre-programmed amount of time as a precision medicine. Thereafter, the device would degrade and be resorbed by the host and a secondary procedure for removal would not be necessary. Using this approach, many of the common adverse effects of pain medication (e.g., gastrointestinal issues of NSAIDs, diversion) would be limited as the API would only be exposed to the implantation tissue and the vasculature in its path to metabolic excretion. In order to properly design such a device, it is important to consider the biological processes that will be aided or impeded, and to ensure that no additional harm or complications will be brought on by the device. These requirements will be contingent upon the (i) polymer used, (ii) API for targeting biological processes, and (iii) overall matrix design, all of which will be discussed in detail in this perspective. Furthermore, direction for an ideal postoperative polymeric device for pain management will be proposed.

## Targeting postoperative pain via APIs

Acute postoperative pain is prevalent in patients following a surgical procedure, which can be broken down into nociceptive (with the physical injury as a stimuli) and inflammatory pain (the biological response to this stimuli, thereafter)^8^. If not treated appropriately, improper healing of the surgical site can occur in which case inflammation becomes chronic or/and neural plastic changes persist, leading to chronic pain^9^. In particular, neuropathic pain is an example of postoperative pain progressing to chronic pain, due to nerve injury after certain types of surgeries, such as thoracotomy^9,10^. Surgery and incision also can cause neurogenic inflammation due to the release of neuropeptides such as substance P and calcitonin gene-related peptide from pain-sensing nerve fibers. Neurogenic inflammation causes edema and contributes to postoperative pain^11^. Understanding the multitude of classifications of pain and the physiology of this process as well as the wound healing process allows the treatment approach to be specified per patient. Several chief mechanisms, such as peripheral sensitization (sensitization of nociceptive neurons in the peripheral nervous system, PNS), central sensitization (sensitization of spinal cord and brain neurons in the central nervous system, CNS), and activation of glial cells (e.g., satellite glial cells in the PNS and microglia and astrocytes in the CNS) have been implicated in the transition from acute to chronic pain and pathogenesis of chronic pain^12–16^.

Different levels of analgesia are required depending on the type, severity, and persistence of pain following a surgical procedure. Devices can be designed accordingly to match and aid each step along these processes. For example, devices that are used solely for patient comfort and recovery following a surgery (i.e., acute pain), should release API efficaciously for a maximum of 14 days, with specific attention to proper analgesia from days 3 to 5^17^ and could possess an agent to aid in wound healing. The contents of the device will depend on the biological processes that are targeted with insertion. Common drugs that are currently used for postoperative analgesia are opioids or nonsteroidal anti-inflammatory drugs (NSAIDs). Local anesthetics (LAs) are more often used pre- or perioperatively but could also be utilized to provide pain block following a procedure^18^. Other classes of drugs may be used singly or as adjuvants for other types of pain or complications that arise following the procedure (e.g., gabapentin, acetaminophen, tramadol, anticoagulants, or other symptom reducing medication)^19,20^.

### Opioids

**Fig. 1:**
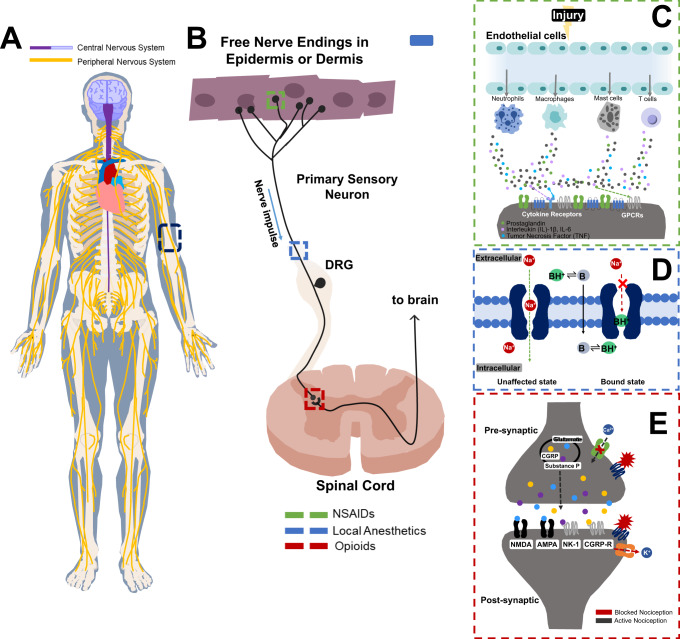
Various analgesic agents can be added to an inserted polymeric matrix to alleviate pain following a surgery. Selection of the API will depend on the targeted biological process that is intended to be aided or impeded. Pain is a neurological process and as such, analgesic APIs act on the central and peripheral nervous system (**A**). Each of three classes of analgesic drug acts upon a site or multiple sites of the pain pathway to block or mitigate the signal transduction necessary for pain (**B**). NSAIDs act on the cyclo-oxygenase (COX) enzyme to block the production of inflammatory mediators, such as prostaglandins (e.g., PGE_2_), as well as interleukin (IL)-1, IL-6 and tumor necrosis factor (TNF), that are responsible for the initiation of nociception (**C**)^142^. Local anesthetics block ion channels in the periphery to create an inactive state, preventing the signal from continuing (**D**). Opioids work on the central and peripheral terminals and spinal cord and brain neurons to suppress pain signaling. Their mechanisms of action involve the G-protein-coupled receptors in nociceptive neurons in the PNS and CNS to inhibit the influx of calcium ions via calcium channels on primary sensory neurons, leading to an inhibition of neurotransmitter release and blockade of pain transmission^143^. In postsynaptic neurons in the CNS (e.g., spinal cord dorsal horn), opioids also activate potassium channels and create an influx of potassium ions to hyperpolarize neurons, therefore, decreasing their activities (**E**). DRG (Dorsal Root Ganglion), B (unprotonated local anesthetic/base), and BH^+^ (protonated local anesthetic/base).

Opioids are among the most potent analgesics but possess risks associated with their mechanism of action. Opioids, such as morphine act upon G-protein-coupled receptors on the nerve terminals and neuronal cell bodies in the PNS and CNS to suppress neuronal activities (Fig. 1E)^21^. Opioid receptors, including μ, δ, and κ opioid receptors, are found on primary sensory neurons in the PNS^22,23^ as well as neurons in the CNS^21,24^, allowing them to act in different dispersed physiological manners. Morphine’s analgesic and adverse effects (e.g., addiction) are mediated by μ opioid receptors^25^, although recent studies also suggested some side effects are mediated by β-arrestin-2 and the biased agonists may produce analgesia with less side effects^26^. Patients can build a tolerance to opioids, causing them to increase doses as needed and heighten the risk for dependence and abuse^27^. Opioids were also shown to produce paradoxical hyperalgesia in humans^28^. Preoperative opioid use is associated with clinically relevant worse knee functions in a 2-year report from knee surgery patients^29^. In particular, ingesting opioids can lead to respiratory depression, which if taken at high enough, doses can be lethal, leading to the ongoing “Opioid Crisis” in the US^30^. Therefore, the use of opioids should be limited if not avoided all together.

### Local anesthetics

LAs are typically used for quick pain relief or to induce numbing. They are commonly used prior to surgery to dull any pain sensations to the operational site, especially in dental surgery^31,32^. The key mechanism of action of LAs proceeds through blockade of neuronal voltage-gated sodium channels (Nav), consisting of nine subtypes (Nav1.1–1.9). The Nav1.7–Nav1.9 subtypes are expressed by nociceptive sensory neurons in the PNS, and therefore, selective blockade of these subtypes, especially Nav1.7, has been a tremendous industrial effort for developing pain therapeutics by avoiding Nav1.5 which is expressed on heart cells^33,34^. LAs bind to the pore region in the ion channels (Nav1.1–Nav1.9) and block Na^+^ influx into the neuron, blocking action potential firing and causing nerve conduction blockade (Fig. 1D). LAs, such as lidocaine and bupivacaine, are often better used for intra- and preoperative analgesia and can shorten patient stay and lower the need for postoperative analgesia^35^. Notably, the CNS-related side effects such as respiratory depression and risk of death were shown to be associated with poor LA dosing, especially in combination with sedatives and opioids^36^. Recent experimental efforts have also been made for blocking specific afferent fibers using LAs. QX-314 is a cell membrane-impermeable lidocaine derivative, and it can only block sodium channels following intracellular delivery. Interestingly, co-application of capsaicin and QX-314 enables delivery of extracellular QX-314 to pain-selective C-fibers that express capsaicin receptor transient receptor potential subtype V1 (TRPV1), causing QX-314 entry through TRPV1 channel for pain blockade^37^. Furthermore, co-application of QX-314 with flagellin, a bacterial component that activates Toll-like receptor 5 (TLR5), enables delivery of extracellular QX-314 to touch-sensing A-fibers, leading to a selective blockade of mechanical allodynia (pain induced by normally innocuous mechanical stimulation such as light touch), a cardinal feature of chronic pain^38^. Although these QX-314 based approaches are promising in preclinical studies, the neurotoxicity profiles of QX-314 in different nerve fibers remain to be tested in clinical studies.

### Nonsteroidal anti-inflammatory drugs

NSAIDs are a class of analgesics that can alleviate pain and reduce inflammation at the site of injury. NSAIDs are enzyme inhibitors that target cyclooxygenases which are responsible for the production of prostanoids (e.g., thromboxane (TXA_2_), prostacyclin (PGI_2_), and prostaglandins). Prostaglandins are inflammatory mediators that regulate inflammation, control blood pressure, and are involved in the contraction and relaxation of both smooth muscle and blood vessels, while thromboxane causes platelet aggregation and is important for thrombosis. Prostaglandin E_2_ (PGE_2_) is the chief prostaglandin for pain regulation and induces inflammatory pain by binding to its receptors (EP1–EP4, especially EP4) and causes activation of protein kinase A (PKA) and tetrodotoxin (TTX)-resistant sodium channels (e.g., Nav1.8)^39–41^. PGE_2_ also contributes to acute to persistent pain transition by activation of epsilon isoform of protein kinase C (PKCε)^42^.

Two isoforms of the COX enzyme are known, COX-1 and COX-2, which differ in regulatory function, conversion mechanism, and biological region in which they act^43^. COX-1 is constitutively expressed and responsible for the conversion of arachidonic acid to prostaglandins that maintain homeostatic roles, such as maintaining the lining of the gastrointestinal tract and regulation of platelet aggregation. COX-2 on the other hand, is highly inducible after tissue injury and inflammation^44^ and responsible for the production of prostanoids (PGE_2_) that are involved the induction of the pain signal and overall inflammatory response following an injury amongst other roles (Fig. 1C). Highly selective COX-2 NSAIDs are preferred due to their lack of interference in normal functioning and a decreased risk of GI side effects while still providing the appropriate analgesia and therapeutic effects.

Many other APIs could be beneficial as components of a postoperative pain management device, though the list of classes mentioned covers the most commonly used medications. Application as well as compatibility considerations will be what dictates the addition of specific molecules into the polymer matrix, with special focus on three classes of analgesics used for postoperative pain, as described in Fig. 1. Below we will discuss the strategies that have already been developed and highlight how to improve upon these methods with the goal of achieving efficacious pain management following surgery. With the advent of controlled release devices, high degrees of tunability are achievable and, thus, a suite of potential pain management treatments are possible.

## Possible administration methods for postoperative pain management

Current postoperative pharmacotherapy is typically achieved by dosage forms that make the entirety of the dose immediately bioavailable. These methods are often associated with fluctuating plasma concentrations, systemic adverse effects, and limited control on the regional distribution of drug. On the contrary, controlled drug delivery provides the delivery of moderate doses safely to the specific area of injury. Controlled release systems include devices that can provide analgesia via multiple different administration methods such as injectable, transdermal, or subcutaneous routes.

### Oral dosage models

Each administration method and modality currently in practice for the controlled delivery of APIs is an invaluable medical tool. The least invasive, most popular method affording the greatest patient control method is oral delivery. Oral pills/tablets/capsules can be formulated using various methods, such as compression or injection molding^45,46^, 3-D printing^47,48^, extrusion^49,50^, and coating^51^ to name a few. In these systems, an API is typically homogenously mixed into a matrix of controlled release agent(s) to be released via diffusion or degradation of the matrix when introduced to an external environment.

**Fig. 2:**
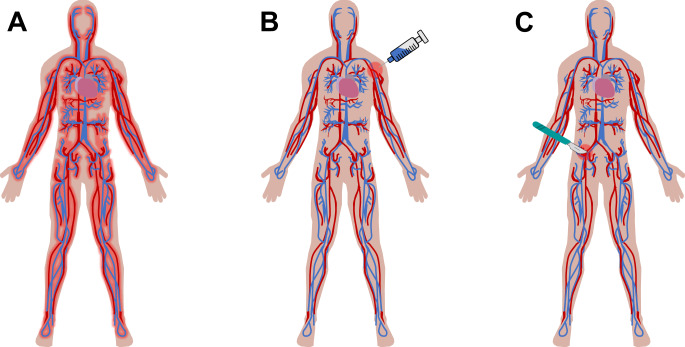
Schematic of the circulatory system to depict different drug distribution effects from various administration methods. **A** Oral administration is the most common and simplest method for analgesic delivery to date, however, first pass metabolism limits bioavailability to the active site and exposes even healthy tissue to the drug, therefore making it inefficient and uneconomical. Both (**B**) injectable (mainly subcutaneous or intramuscular) and (**C**) implantable controlled delivery methods afford a more localized exposure of the drug when administered, especially when impregnated into a controlled release matrix. If implanted at the site of injury, therapeutic effects should stay localized to a radius relative to the site of insertion, which will depend on the system itself (i.e., drug solubility and absorption).

MSContin®, an extended-release oral dosage form of morphine sulfate, is an example of a controlled release formulation that has shown promising results in comparison to noncontrolled methods^52^. In this method, morphine sulfate is blended in both hydrophilic hydroxypropyl methylcellulose and hydrophobic hydroxyl ethyl cellulose in order to provide both rapid and sustained release, respectively. Many other commercially available oral dosage forms for the delivery of opioids also exist. However, the oral administration route has the potential for diversion and adverse effects due to systemic exposure of the opioid as well as increased dose requirements over time (Fig. 2A). Many APIs are not suitable for oral drug delivery due to degradation or other changes from the acidic environment of the stomach or intestines^53^, first pass metabolism^54^, or other compliance issues with processing. Localized delivery of drug directly to the injured site has been introduced to solve some of these issues.

### Injectable controlled release methods

Injectable delivery bypasses some of the issues that arise with oral delivery models such as the first pass metabolism and degradation. Injectable systems have been explored as localized delivery devices in the form of both gel matrices and nano- or microparticles^55,56^. If used with a controlled delivery matrix, these methods have the potential to maintain localized drug delivery and therefore enhance bioavailability (Fig. 2B).

To develop these systems, particles, liposomes, or emulsions have been suspended in a biocompatible medium or a degradable gel to achieve control of the drug delivery and to prevent diffusion of the particles away from the injection site^57^. Injectable doses are a minimally invasive and a quick form of controlled delivery. Given their high surface area, release is often fast, though can be prolonged depending on the fabrication method^58^. Nevertheless, this route introduces some disadvantages, such as sterility and storage stability, invasive nature, and could require administration by a physician or nurse. Limited by their size, nanoscale devices run the risk of systemic distribution when dosed intravenously. This hurdle has led to the development of targeted drug delivery, which is most often used in cancer research as chemotherapeutics are particularly detrimental to healthy tissue^59^, or incorporation of the particles into a more controlled media.

### Transdermal controlled release methods

Transdermal delivery systems eliminate the invasive nature altogether and provide a route that sidesteps gastrointestinal drug degradation. Transdermal delivery systems have been implemented with lidocaine in the form of pain-alleviating patches for post herpetic neuralgia^60^ and capsaicin for treatment of post-shingles nerve pain^61^. These systems are intended to relieve soreness or pain at a specific area for up to 3 h and 3 months, respectively. In a similar product, Duragesic® (Janssen Pharmaceuticals, Inc., Beerse, Belgium) is a fentanyl-containing reservoir transdermal delivery system that delivers the potent analgesic for 72 h^62^. Fentanyl dosing is controlled by surface area of the patch that is applied. However, this device is only recommended to patients who have moderate to chronic pain that cannot be treated by other medications and who require continuous analgesia so as not to introduce the serious adverse effects associated with opioids. Although a promising administration method, most transdermal methods are limited by issues of drug solubility and the ability of the API to bypass the skin barrier. To bypass these pitfalls, the effectiveness of external stimuli (e.g., iontophoresis, ultra sound, or microneedles) to increase efficacy drug release^63,64^ has been explored. Additionally, a lag time has been associated with transdermal mechanisms, which introduces inefficacious delivery of drug to the patient initially as well as the potential for adverse effects to persist after patch removal^65^. Although transdermal delivery systems do deliver the drug to a localized area, this method has disadvantages, such as those associated with Lidoderm® (Endo Pharmaceuticals Inc., Malvern, PA)^66^ and Duragesic®, that make it a non-optimal route for postoperative pain management.

### Implantable controlled release methods

The most invasive, yet effective method for achieving localized drug delivery is the use of implantable devices. Both active (require an external trigger) and passive (e.g., diffusion) implantable drug delivery systems have been reported in the literature, and offer various levels of control over drug delivery and durability of the device^67^. Implantable administration is beneficial in comparison to other routes as it makes the drug directly available to local tissue, without any physical barriers (i.e., skin), and implants are generally large enough so systemic distribution of the device is not an issue.

Depending on the intended length of therapy, biodegradable and non-biodegradable devices have been used. Non-biodegradable polymers (silicones, polyurethanes, polyacrylates, and poly(ethylene vinyl acetate) are often used for prolonged applications, such as contraception to provide longer release and require removal once the release period is over^68^. An invasive secondary removal procedure would negate the purpose of the pain-alleviating implant and therefore, biodegradable polymers for controlled pain management devices would be ideal. In any biodegradable implant, API is released directly into tissue for therapeutic effects and is governed by diffusion (spontaneously or triggered) from the matrix or degradation of the material (Fig. 2C)^65,69,70^. Biodegradable polymers can also be tailored to degrade over a long period of time, offering a long duration still for therapeutic relief (e.g., up to a month). However, long-term implantation could trigger adverse effects, such as formation of a biofilm and infection, so the safety of the implant over time must be demonstrated.

Administration route of a postoperative device will depend on the polymer material as well as specific patient considerations (e.g., types of pain or other biological complications, volume of surgical site, and time frame after surgery). Although some systems will be more ideal for localized drug delivery, they might not be plausible given these considerations. Therefore, it would be beneficial to have a material that has the mechanical flexibility to be fabricated for multiple routes of administration. Below, we highlight the current devices available that display the potential to be used as postoperative pain management systems and suggest areas for improvement.

## Design parameters of an ideal controlled delivery device

Currently, the most widely used and available clinically applied non-opioid controlled drug delivery system for postoperative pain management is Exparel® (Pacira Biosciences, Inc., San Diego, CA). Since 2012, more than 7 million patients have received this treatment following a surgical procedure^71^. Exparel® is a single-dose injectable suspension that utilizes DepoFoam® technology to encapsulate bupivacaine into a liposome via double emulsion to safely provide anesthesia to the injured tissue^72^. It is available in a 13.3 mg/mL dose and offered in a 10 and 20 mL single-dose vial, but can be expanded with a saline solution for larger surgical sites.

Liposomal bupivacaine is able to provide lower or equal systemic drug levels (*C*_max_) at higher doses and prolonged analgesia (*T*_max_) in both animal^73^ and human^74^ trials. Importantly, Exparel® limits the need for opioids following surgery by properly providing analgesia directly to the surgical site^72^. Proper controlled delivery is achieved with these devices in comparison to free bupivacaine, limiting the systemic toxicity, and therefore adverse effects, while lowering pain scores in patients^75^. Notably, in a clinical study with 352 patients, postoperative complications related to the Exparel® interscalene block were observed in 58 patients (16.5%), including 21 major complications (6.0%) that required emergency department visits^76^. Additionally, although systemic toxicity is limited, multiple, closely spaced injections of the liposomes are needed to administer an efficacious dose. Given this, it would be beneficial to engineer a device that could extend the delivery of a sufficient dose following a single application while maintaining efficacy.

**Fig. 3:**
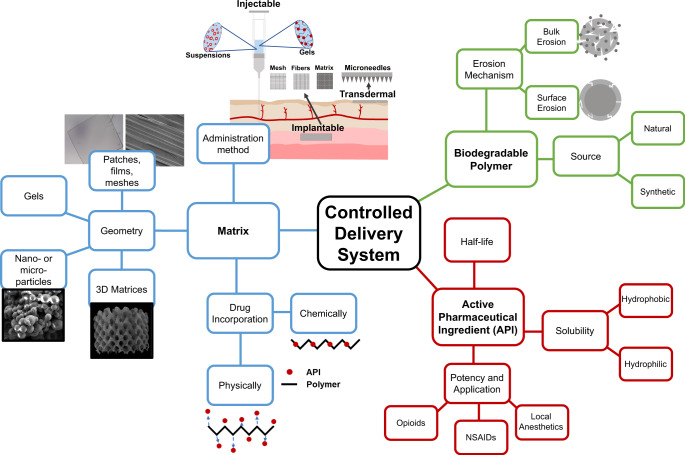
Polymeric drug delivery systems afford many different size scales due to the flexibility in processing of synthetic polymers. Each scale class offers it’s own benefits and application methods that prove useful for targeting specific types of pain.

When developing a controlled release formulation, there are three main elements to consider: (1) the combination of the components into the matrix, (2) the choice of API, and (3) choice of biodegradable polymer (Fig. 3). The choice of API is dependent on the other parameters and recent efforts in the literature are summarized in Table 1. API’s can be used singly or in combination depending on the nature and location of the injury. While the search for more potent and specific APIs for pain management are ongoing, the library of available compounds is fairly significant.

**Table 1:** Polymeric controlled drug delivery devices organized by polymer class, duration of in vitro release, then duration of analgesia exhibited in vivo, if available.

Polymer class	Polymer system	Administration method	Analgesic/anesthetic	Max duration of in vitro release	Max duration of analgesia	Reference
Natural	Collagen	Cross-linked sponges	Piroxicam	10 h	N/A	^144^
	CARR and HA	Lyophilized wafer	Lidocaine and AgNPs	6 h	N/A	^145^
	Gelatin/alginate	Transdermal bioadhesive	Bupivacaine and Ibuprofen	3 Days	N/A	^146^
	HA-Drug conjugates	Subcutaneous injection	Morphine, Codeine, Naloxone	0.1–55 Days	N/A	^89^
Anhydrides	P(SA:RA)	Gel	Bupivacaine HCl	10 Days	30 h	^147^
	FAD-based poly(anhydride)	Cylindrical implant	Bupivacaine HCl	25 Days	N/A	^106^
	pSA	Nanoparticles	Ropivacaine HCl/Ropivacaine	1 Day/6 days	N/A	^108^
Ortho esters	POE IV	Viscous gel	Mepivacaine HCl	N/A	4 h	^86^
Esters	mPEG-PLGA	Hydrogels	Ketorlac	48 h	18 h	^148^
	PLGA-PEG-PLGA	Gels/microsphere	Bupivacaine and dexmedetomidine	9 Days	37 h	^133^
	Lipid-based PEG and PCL	Nanoparticles	Ropivacaine	6 Days	1.5 Days	^149^
	PELA	Nanoparticles	Ropivacaine	N/A	3 Days	^150^
	TDP-PEG	Injectable systems	Tetrodotoxin	30 Days	3 Days	^151^
	PET	Meshes	Ropivacaine	60 min (burst effects)	4 Days	^152^
	PLA/PLGA	Conjugated Nanoparticles	Fentanyl	10 Days	6 Days	^153^
	PLGA	Sheets	Lidocaine	2 Weeks	1 Week	^154^
	PLGA	Coated stainless steel implant	Ketorolac and lidocaine	30 Days	4 Weeks	^155^
	PCLA-PEG-PCLA	Injectable gel	Celecoxib	100 Days	4–8 Weeks	^156^
	PLGA	Microfluidic implant	Bupivacaine HCl	4–8 Days (media dependent)	N/A	^157^
	PEO/PVA-SA	Double-layer nanofiber scaffold	Gabapentin/acetaminophen	1 h/10 h	N/A	^158^
Amides	PNVCL	Thermosensitive gels	Acetamidophenol and etoricoxib	1 Day	1 Day	^159^
	PEA	Injectable microspheres	Celecoxib	80 Days	1 Week^a^	^58^
	PNIPAM	Hydrogel	Buprenorphine	7 Days	N/A	^160^

*CARR* carrageenan, *HA* hyaluronic acid, *SA* sebacic acid, *RA* rineloic acid, *FAD* fatty acid dimer, *POE* poly ortho ester, *PEG* poly ethylene glycol, *PLGA* poly lactic-co-glycolic acid, *PCL* poly caprolactone, *PELA* poly ethylene oxide/poly lactic acid, *TDP* poly triol dicarboxylic acid-co-poly ethylene glycol, *PET* Polyethylene terephthalate, *PLA* poly lactic acid, *PCLA* polycaprolactone-co-lactide, *PVA* poly vinyl acetate, *PNVCL* Poly N-vinylcaprolactam, *PEA* poly ester amide, *PNIPAM* Poly N-isopropylacrylamide.

^a^Anti-inflammatory response measured only, measured as production of PGE2 in rat model.

Not only does the composition of the API effect the overall impact of the device, but the manner in which the API is embedded into the device also plays a significant role. An important design element in controlled drug delivery devices is drug incorporation through physical or chemical means. The synthetic nature of most polymers affords the ability of drug to be linked onto the polymer backbone upon synthesis and later to be cleaved hydrolytically or by other methods^77^. As one could imagine, release from these devices is dependent upon the cleavage of built-in labile bonds to release the therapeutic molecule. Physically imbedded drug, on the other hand, can be mixed into the system and held in place by intermolecular forces (i.e., ionic, hydrogen bonding) or steric interactions. In these systems, energetic considerations of the environment are important, including the release media, physical agitation, temperature, and pH used to promote the diffusion of the drug out of the matrix. Importantly, at the site of tissue injury, the pro-inflammatory environments have lower pH due to increased proton levels and acidosis, which will have an impact on the API elution; low pH is a hallmark of injured tissue. Some drugs show pH-sensitive binding to their targets. For example, a nontoxic pain killer has been developed to activate peripheral μ-opioid receptors at the source of pain generation. It produced injury-restricted analgesia in animals with pathological pain without exhibiting respiratory depression, sedation, constipation, or addiction potential of opioid^78^. Moreover, inflammation is known to upregulate proteases, such as matrix metalloproteases that are implicated in the pathogenesis of pain^79^. Some collagen, gelatin, and peptide-based polymers may be sensitive to proteases in the inflamed tissues.

**Fig. 4:**
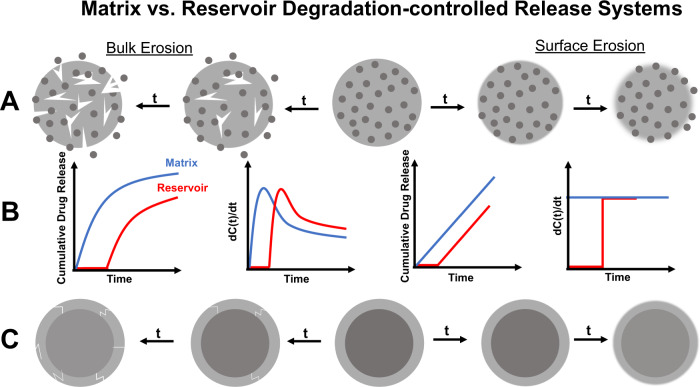
Degradable polymeric systems can erode by different mechanisms depending on their chemical structure and mechanial properties. This factor, along with how the drug is incorporated into the matrix will ultimately impact how drug elution from the system occurs. Controlled delivery systems can incorporate drug in a matrix (**A**, **B**) or a reservoir (**B**, **C**) system. Depending on the application, this will alter the drug release from the system in a specific manner. Reservoir systems are characteristic of a delayed release as the polymer surrounding the drug core degrades whereas matrix systems will depend more highly on the polymer-drug interactions among other factors (**B**). If solely based on polymer degradation, however, the release will either be more exponential (bulk erosion) or continual (surface erosion). Bulk erosion usually provides the system a “burst” release at early time points, followed by a continuous decrease in rate.

API can also be stored in a matrix in a reservoir or dispersed manner. In a dispersed system, drug is homogeneously distributed throughout the polymeric matrix (Fig. 4A) whereas in a reservoir, drug is confined to one location (Fig. 4C). When drug is mixed within the matrix, system swelling, drug diffusion, polymer degradation, or outside stimulus can impact the manner in which drug elutes from the system. Alternatively, drug release is dictated by the ability for the drug to penetrate the outer layer, the polymer degradation, and/or the diffusion of the drug to the surrounding environment, usually resulting in a delayed release with reservoir systems. The impacts of these considerations on API elution is depicted in Fig. 4B.

Overall release, regardless of drug distribution, also depends on the geometry of the drug/polymer conjugate. Mainly, it is impacted by the surface area to volume ratio (SA/V)^80^, or, the size scale on which the system can be described (e.g., nano, micro, or macro). Size scale of the considered polymeric device impacts the release rate, total dose of the system, and application methods possible. For example, there has been significant interest in current literature surrounding the development of nano-technology^81^. Working on a micro- to nano- scale for drug delivery affords the ability to achieve a minimally invasive and rapid drug delivery system. Explicitly, due to high surface area and minute size, drug elution is typically rapid and dependent on degradation or diffusion^80^.

Alternatively, increasing the size of the device also increases the potency or the maximum dose of the system. As an example, thin films can be solid or be comprised of a fibrous or grid structure, where drug can be encapsulated or trapped (i.e., intramolecular forces) within the matrix. Release can be tuned accordingly with the system: fibers offer a lower barrier and higher surface area for drug to diffuse through, and thus often affords a quicker overall release when considering SA/V. Solid film matrices on the other hand, contain drug that is mixed within the confines of the polymer and must diffuse out in accordance with drug solubility or matrix swelling. Gels also offer a different release matrix geometry and also mechanical properties that could be beneficial as an implantable or injectable method of administration. Larger scale implants are also presumable and afford more flexibility with implant shape and increase resolution, though, will be more difficult to implement due to their size constraints.

The last design element for controlled delivery system is the degradable polymer used as the matrix. Degradable polymers can be classified into two main categories: natural and synthetic. Natural biopolymers are sourced from extracellular matrix or plants and synthetic polymers are often derived from man-made precursors and are not naturally occurring. Between the two classes, a wide variety of materials are available for use in controlled drug delivery devices for pain management. Polymers have been used widely in medicine for nearly 60 years^82,83^. As materials and synthetic methods have evolved, the resulting properties of these materials have been able to meet an increasing number of unmet medical needs. While the vast majority of polymers used in medicine are not degradable, they require secondary removal when used in drug delivery applications and their elution properties are solely diffusion based. Over time, the API banks are exhausted or they will likely become non-efficacious as drug could be trapped in matrix core.

On the contrary, degradable polymers are gradually resorbed by the body upon insertion and long-term complications can be avoided. When considering degradable polymer matrices, different release profiles are imaginable as induced by polymer degradation, matrix swelling, and drug diffusion. Elution profile, as well as many other device behaviors are highly dependent on the chemical composition of the polymer backbone and thus the mechanical and structural properties thereafter. The versatility of such factors has led to many biodegradable materials that have been proposed for drug delivery applications.

The degradation mechanism is crucial in considering a polymeric device for a specific drug delivery application. In a surface eroding material, degradation occurs from the exterior surface and is usually comprised of labile bonds that get exposed at the surface, but none in the bulk of the material^84^. Surface eroding polymers, have consistent release rates that are proportional to the polymer degradation (Fig. 4). A few examples of polymers in this category that have already been utilized for drug release are polyanhydrides^85^ and poly(ortho esters)^86^.

Bulk erosion occurs throughout the entirety of the material^84^. As such, these polymers tend to be more hydrophilic than surface eroding materials, thus allowing for water to penetrate and degrade the entirety of the material. Drug release from these systems is often dependent upon diffusion, swelling, or degradation of the polymer. Bulk degradation can cause pores to form in the material through which the drug can diffuse, causing an increase in release rate. However, the duration of degradation can be considerably long for these polymers if they are hydrophobic, and drug release is limited by the ability of the drug to diffuse out of the polymer or the ability of the matrix to swell. As such, labile bonds, copolymers, or other formulation controls are imparted to increase the drug release. Bulk eroding polymers include polyesters and polyamides^87^. Below, we will discuss the classes of polymers that have been implemented for controlled pain management with specific applications.

### Natural polymers

Perhaps the simplest implication of biodegradable biomaterials has been the development of natural polymers. Biologically derived systems are generally considered to be less risky as they will degrade into materials that the body can readily metabolize. As such, natural polymers or conjugates have been analyzed for their ability to deliver anesthetic or analgesic molecules for controlled pain management. These include, but are not limited to, proteins (e.g., collagen, fibrin, and silk) and polysaccharides (starch, alginate, gelatin, chitin/chitosan, and hyaluronic acid derivatives)^88,89^. Some recent examples of controlled delivery devices that implement natural polymers for therapeutic analgesia are highlighted in Table 1.

In the last few months, Irish pharmaceutical company Innocoll Holdings Limited, received US FDA approval for Xaracoll® with indications for acute postsurgical pain relief for up to 24 h in adults following open inguinal hernia repair. The approval route was a 505(b)2 pathway using a new delivery system for a well-characterized drug. Xaracoll® is a non-injectable drug-device combination in the form of a fully bioresorbable collagen implant containing bupivacaine hydrochloride. Xaracoll® is placed directly into the surgical site during surgery and, after placement, releases bupivacaine sustainably^90,91^. The collagen is then resorbable or can be remodeled over time.

In the realm of drug delivery, natural polymers usually only release drug efficaciously for a few hours to a day. This duration would be considered too short to enhance patient healing and recovery following a procedure. Therefore, synthetic polymers are often used to lengthen the duration of analgesia or provide more robust mechanical properties.

### Synthetic polymers

Synthetic polymers are often used to sidestep the durability complications that can be seen with natural polymers. Most novel polymers used in biomaterial devices report limited cytotoxicity and inflammatory response. With a variety of synthetic mechanisms and building blocks comes an array of properties that yield drug release profiles that fit specific applications. Although other fields of drug delivery are more numerous, there are a number of examples in the literature of synthetic polymers used for analgesia that could prove useful to combat postoperative pain.

#### Poly(anhydrides)

Langer^92–94^ was the first to develop and implement polyanhydrides intomedical applications. Since then, they have been used as degradable biological matrices and have been used to deliver small molecules^95–97^, proteins^57,98,99^, bioactive agents to promote bone formation^99,100^, and more popularly, chemotherapeutic drugs^101–103^. Polyanhydrides were one of the first materials to gain application-based regulatory approval. Gliadel® Wafer is a polyanhydride material that was approved by the FDA for the delivery of BCNU (bis-chloroethylnitrosourea) directly to the brain to treat glioblastoma multiformae^104^. The labile anhydride bonds in the polymer backbone and overall hydrophobic water transport properties make polyanhydrides matrices surface eroding, and therefore heterogeneously degradable materials^94^. Polyanhydrides are considered one of the most hydrolytically reactive biomaterials. Moreover, by tuning the hydrophobicity of the polymer backbone results in a variation in water penetration rates which yields an array of degradation profiles lasting a week to year^85,105^.

A possible limitation of polyanhydrides for drug release purposes is the reactivity of the polymer with various amines that occurs at high temperatures. The reactivity of the active agent for sustained delivery must be considered prior to introducing it into a polyanhydride matrix. Many analgesic compounds contain reactive amine groups, such as LAs, which may explain the lack of current literature using polyanhydrides for analgesic delivery. Nevertheless, it has been shown that polyanhydrides are capable of delivering LAs via implants^106,107^ and nanoparticle injections^108^. Current literature does not offer evidence of polyanhydride systems for controlled pain management clinically, though the in vitro release profiles exhibit control over the duration of elution. With the proper development, polyanhydrides could be used as a rapidly degrading analgesic device for postoperative pain.

#### Poly(orthoesters)

Another group of surface eroding polymers used in drug delivery are polyorthoesters (POEs). Two major synthetic routes for POEs are available. Originally, these polymers were prepared by a condensation reaction of 2,2-diethoxytetrahydrofuran and a di-alcohol (Chronomer^TM^ and Alzamer®)^109^. This set of polymers undergo rapid degradation due to the production of γ-butyric acid upon hydrolysis. Heller et al. synthesized an improved POE with the reaction of 3,9-bis(ethylidene 2,4,8,10-tetraoxaspiro undecane) (DETOSU) with various di-alcohols^110^. By synthesizing POEs by this method, no acidic byproduct is produced and, thus, degradation does not proceed in an autocatalytic manner which will be more beneficial clinically.

Depending on the nature of the diol used in the synthesis, solid polymers or viscous semisolid materials are obtainable with POEs, leaving flexibility in fabrication methods of drug delivery vehicles. Drug delivery has been exhibited with POEs and appears to follow a predominantly erosion-controlled path^111^. Poly(ortho esters) have been used to delivery small molecules^112^ as well as macromolecules, such as proteins^113^. Moreover, Heller et al. suggested the development of POE gels for postsurgical pain management with the controlled delivery of mepivacaine, as outlined in their review, though more recent examples are lacking^86^.

#### Poly(esters)

The class of poly(esters) has made significant progress as degradable biomaterials due to their tunable degradation via hydrolysis at the ester and/or ester-analogous site. With this very versatile bond as the premise, a multitude of polyesters have been synthesized and used in drug delivery processes; this also allows many drugs (hydrophobic and hydrophilic) to be embedded into polyester matrices. Some of the most notable uses of polyesters in drug delivery are mentioned below, though it should be noted that this list is not exhausted.

*Poly(ε-caprolactone)*. Poly(ε-caprolactone) (PCL) is an aliphatic polyester that a relatively slow degrading material used for drug delivery. The slow degradation of PCL can be attributed to its semicrystalline behavior and hydrophobicity. Under specific environmental conditions, both hydrolytic and enzymatic surface degradation are possible. Given the length of the degradation time, PCL is better suited for long-acting, implantable devices such as Capronor®, a 1-year implantable contraceptive device^114^. Moreover, PCL has a low melting temperature, and exhibits high thermal stability, rendering it useful for processability. PCL has also been combined with a variety of different polymers in order to obtain different mechanical properties and degradation profiles, such as poly(ethylene glycol) (PEG)^115^ and poly(lactic acid) (PLA)^116^.

*Poly(lactic-co-glycolic acid)*. Poly(lactic-*co*-glycolic acid) (PLGA) is one of the most widely used polymers for controlled drug delivery. Both the copolymer and it’s corresponding homopolymers, poly(glycolic acid) (PGA), and poly(lactic acid) (PLA) are biodegradable polymers that were initially studied for their application as surgical sutures in the early 1960’s^117,118^. This application sparked their journey into becoming one of the most widely used resorbable biomaterials, and eventually led to their high prevalence in the controlled drug delivery realm. PLGA sutures have since been fabricated to contain drug for therapeutic as well as surgical application^119,120^.

PLGA has been used to deliver many molecules from large scale, such as proteins and hormones, to small, such as antibiotics^121,122^. By controlling the stoichiometry of PLA to PGA, the hydrophilicity of the material can be changed, allowing for an array of molecules (hydrophobic and hydrophilic) to be incorporated into the polymer matrix in a compatible manner^123^. Controlling the hydrophobicity also imparts control over the degradation and delivery rate. PLGA’s byproducts are readily metabolized via the tricarboxylic acid cycle, though inflammatory responses have been noted and linked to the production of lactic and glycolic acid upon PLGA degradation^124^. In drug delivery applications, PLGA has been fabricated into injectable delivery methods^125–130^ and copolymers with PEG^131–133^. Much of the work available in the search for controlled postoperative pain management devices uses PLGA or derivatives of the material (Table 1). As demonstrated in these works, altering overall composition, molecular weight, and fabrication method used, PLGA can be utilized as a component to achieve an array of analgesic durations and release profiles, though may be limited by the safety of its degradation products^124^.

#### Poly(ester amides)

Poly(ester amides) (PEAs) provide the benefit of having two hydrolytic degradation sites—the ester and amide bonds. As such, they offer great potential as a degradable biomaterial for analgesia applications. In the work highlighted in this review, PEAs can be fabricated in different application methods and have exhibited the ability to be a multicomponent release system with the ability to provide analgesia for the targeted time frame (Table 1).

## Bench to market considerations

The path to commercialization of new drugs for pain control is a high risk, high reward endeavor. In addition to target and mechanism identification challenges, potent APIs often have significant off-target effects and toxicity. Therefore, precise dosing, dose regulation, and predictable pharmacokinetics are paramount. The delivery paradigm has often determined the selection and commercialization of drug candidates. With high risk developmental challenges for new drugs, companies have resisted risk in delivery strategies posed by new entities and new degradable polymers. For this reason, well established polymer systems, such as the polyesters noted above continue to dominate the delivery landscape despite limitations of bulk erosion and burst release challenges that are mitigated in many cases with elegant formulation or multilayer fabrication strategies^134,135^.

Less than optimal control limits API candidates. More precise control over release would open the doors to more potent APIs. Candidate degradable polymers are certainly in the literature, but the risk paradigm limits their use. An ideal degradable polymer system would be surface eroding to facilitate design and dosing, resorbable without inflammation, and would prevent drug crystallization during formulation. New delivery systems and modalities for existing, well-characterized drugs are a pathway to innovation for several companies looking at postsurgical pain management devices. Durect is in advanced stages of approval for Posimir^TM^, a bupivacaine extended-release solution. Posimir^TM^ utilize an impregnated polylactide and caprolactone in a viscous carrier. Designed as an injectable around the surgical site, they report effectiveness of their extended-release formulation for 72 h. They originally applied for approval in 2014 and were rejected by the FDA. As of January 2020, the Anesthetic and Analgesic Drug Products Advisory Committee of the FDA rejected Durect’s application for Posimir™^136^.

Heron Therapeutics recently resubmitted to the FDA an application for ZYNRELEF^TM^, an investigational non-opioid analgesic, that is a dual-acting, fixed-dose combination of the local anesthetic bupivacaine with a low dose of the nonsteroidal anti-inflammatory drug meloxicam. ZYNRELEF^TM^ was recently approved for use within the European Union in 2021. The carrier matrix is a low molecular mass polydioxanone. Low molecular mass polymers are synthesized via step growth polymerization and are challenging to scale commercially. ZYNRELEF^TM^ is the first extended-release local anesthetic to demonstrate in Phase 3 studies significantly reduced pain and opioid use through 72 h compared to bupivacaine solution, the current standard-of-care local anesthetic for postoperative pain control^137^. These new systems were submitted as FDA 505(b)2 submissions. If successful, the delivery systems will certainly be applied to other drugs.

## Regulatory pathways

The most common regulatory route for a new API for postoperative pain management would be through the US Food and Drug Administration (FDA) Center for Drug Evaluation and Research (CDER) as a drug. There are several designations for this pathway which includes New Drug Applications (NDA) and Abbreviated New Drug Applications (ANDA). In simple terms, NDA’s are for new drugs that have not yet been approved for specific indications and ANDA’s are for generic products.

NDA, also called 505 (b)(1), is the format that manufacturers use to bring a formal proposal to the FDA that a new drug should be approved and made available for use by patients in the United States. The NDA includes a significant amount of information about the drug being evaluated including the precursors, supply chain, synthetic methodology, quality systems, the results of preclinical (animal model) studies, clinical trial results in humans, sterilization methods, efficacy, and even how it will be packaged and labeled^138^. An NDA submission of a new entity, while very lucrative if approved, takes a great deal of time and resources to complete all the necessary requirements to the FDA for review.

ANDA is used to gain approval for a generic version of a drug that has been approved previously and is on the market. Earning approval through an ANDA pathway involves the manufacturer providing evidence to the FDA that the generic product is substantially equivalent to the currently approved product through analytical chemistry and bioequivalence evaluations. The approved indication, dose, and route of administration for the generic will be nearly identical as the reference (non generic) product. The pathway is abbreviated because preclinical and clinical trials are not required as they were performed by the original manufacturer of the product. This route is faster and generally less expensive than a NDA submission.

However, there is an additional pathway that is a hybrid between the and NDA and ANDA application known as 505(b)(2). The pathway was created in 1984 and was created to help avoid unnecessary duplication of studies already performed on a previously approved drug. The law allows a manufacturer to submit their product for FDA review by including data and/or study results originally collected by another manufacturer or researcher if it is available in the public domain. The 505(b)(2) pathway allows manufacturers to submit for FDA approval without performing all the work that’s required with an NDA. These APIs are not strictly generics, but are generally molecular entities that are well defined and have been used extensively in patients. 505(b)(2) can be an option for API’s with a new indication, dosage form, delivery system, or combination with other product or device. While the number of NDA approvals that utilized the 505(b)(2) pathway fell from 75 in 2018 to 64 in 2019, 505(b)(2) NDA approvals continue to make up more than half (56%) of all NDA approvals through CDER^139^. The FDA website shows the average approval time for a 505(b)(2) in 2019 was 10 months. The increasing utilization of this pathway will result in new polymeric delivery systems available within the commercial market. With success and increased confidence and understanding in these systems, the diversity of controlled release properties will enable the formulation of additional APIs that were previously thought to be to potent for use.

## Conclusions and future directions

Although substantial research has gone into the use of biodegradable polymer matrices as therapeutic devices for pain management, current evidence available for novel postoperative pain management methods leaves much to be desired. The works highlighted in this review should act as steppingstones in the development of a device that affords controlled and individualized pain management to patients. Ideally, a device should (i) be biodegradable within a specific pain-classification period, (ii) provide efficacious drug delivery during this period, (iii) involve an administration method that is well-suited for a specific application, and (iv) be easily tuned or personalized per patient. Matrix geometry will be necessary to consider per administration method, but duration and dose of analgesia afforded per the device will be dictated by the polymer (e.g., erosion mechanism and intermolecular interactions or chemical linkage with the therapeutic). Additionally, API will be an important consideration depending on the targeting biological processes (e.g., pain, inflammation, etc.).

There will always be room for improved materials composed of safe and resorbable building blocks. Many of the current polymers used for biomedical devices today are non degradable, most of which will cause complications following insertion. Although they have all worked traditionally, with the advancement of technology needs to come a push towards sustainability and biological safety. Moreover, it is difficult to be satisfied with the available biodegradable polymers in terms of overall performance, especially considering degradation byproducts. Polymers that afford more control over release profiles will enable the use of more potent APIs with a more diverse exhibition of pharmacokinetics and blood half-life. More potent APIs will afford smaller devices with longer duration of analgesia. Designing a polymeric drug delivery device is clearly a multifaceted project, and, alike current biomedical devices, will likely run into the hurdle of consistency in behavior (i.e., drug elution and material degradation) and reproducibility. Nevertheless, overcoming these kinds of issues would greatly benefit this community.

Sustained pain relief for a duration of >24 h is required for effective control of acute pain after surgery. Notably, the incidence of developing chronic pain after major surgeries such as thoracotomy, mastectomy, and amputation is high, and the severity of acute pain is one of the best predictors of chronic pain development^9^. Chronic pain following central sensitization is widespread and associated with emotional stress and posttraumatic stress disorder^140,141^. Given the variety of systems already available, the transformation of current practices for postoperative pain management to safer and more practical and efficacious methods via degradable polymeric vehicles is highly demanded for multiple short-term and long-term benefits.
